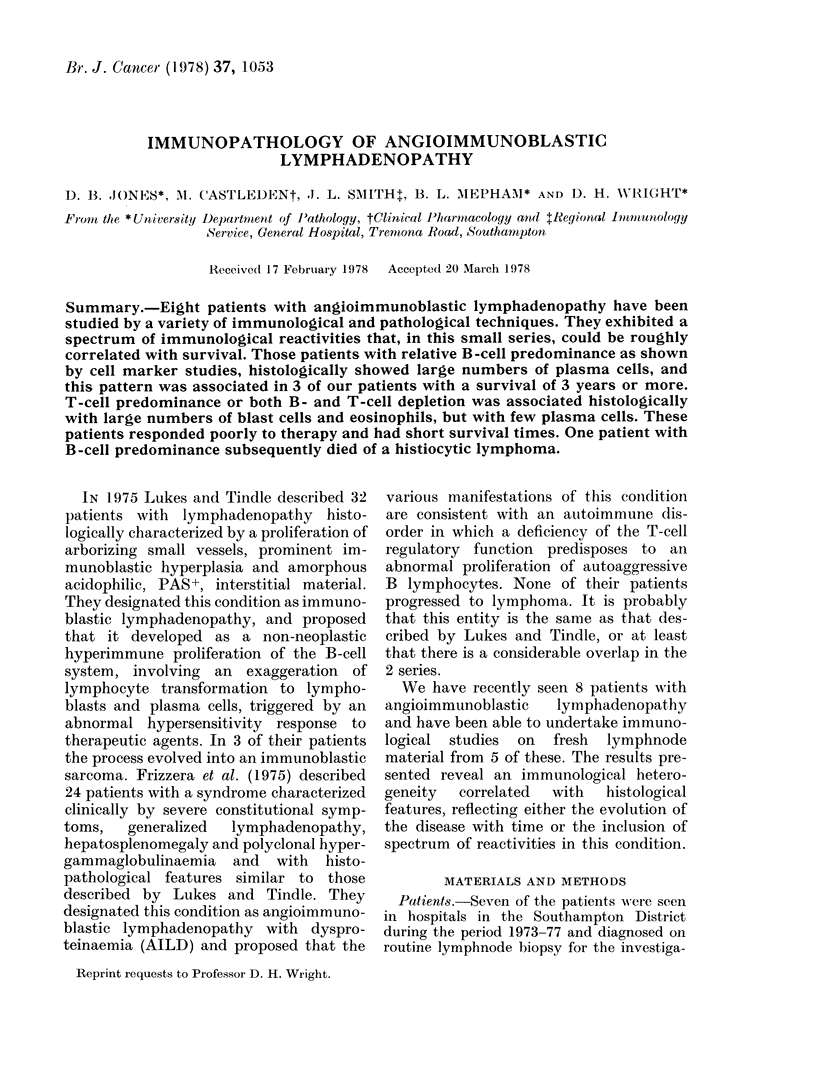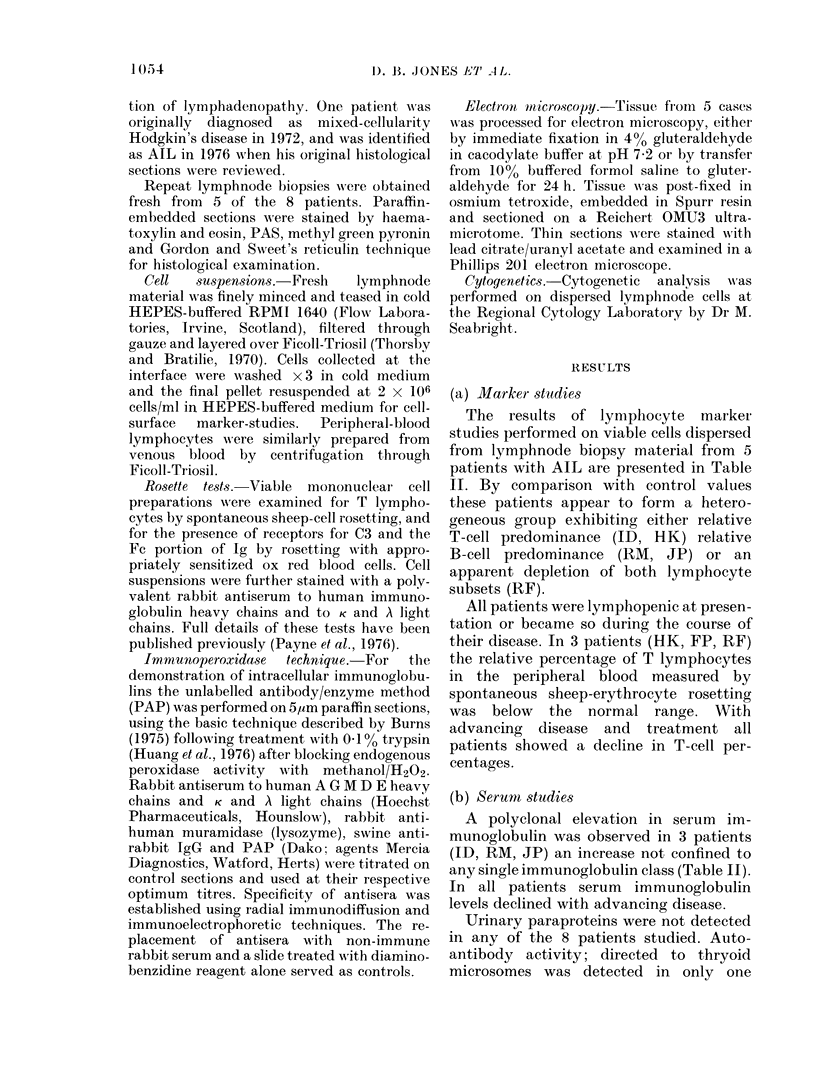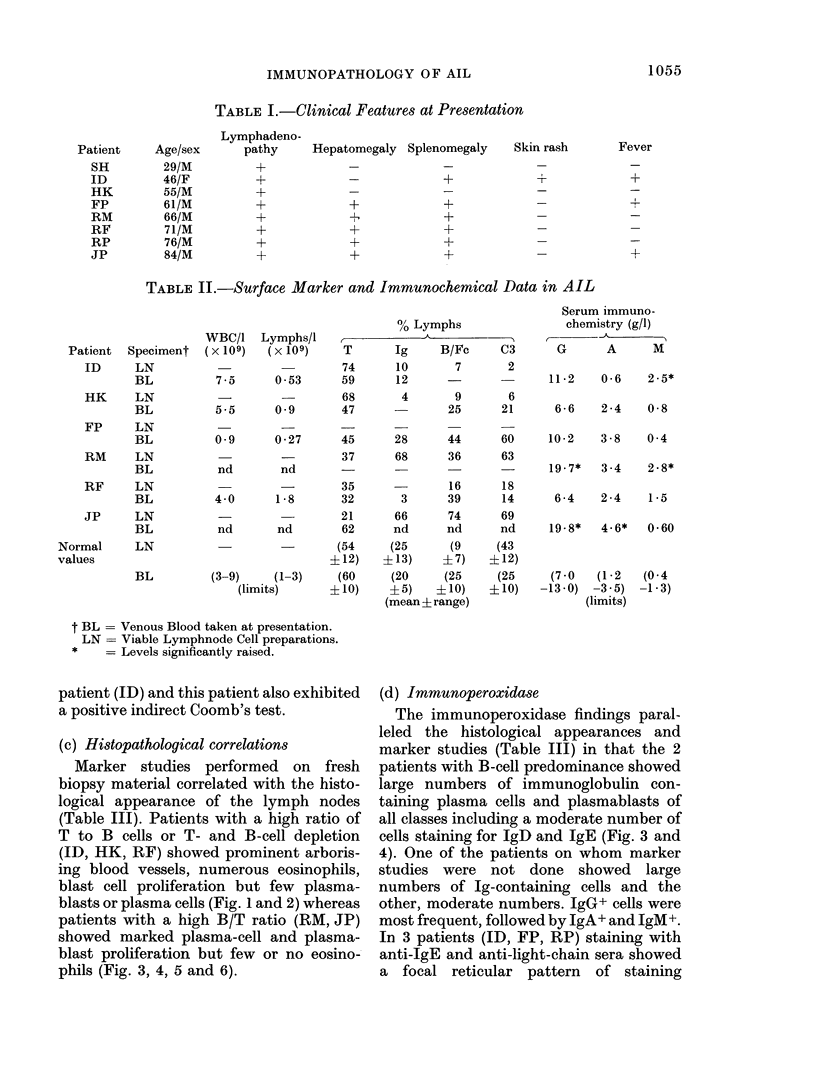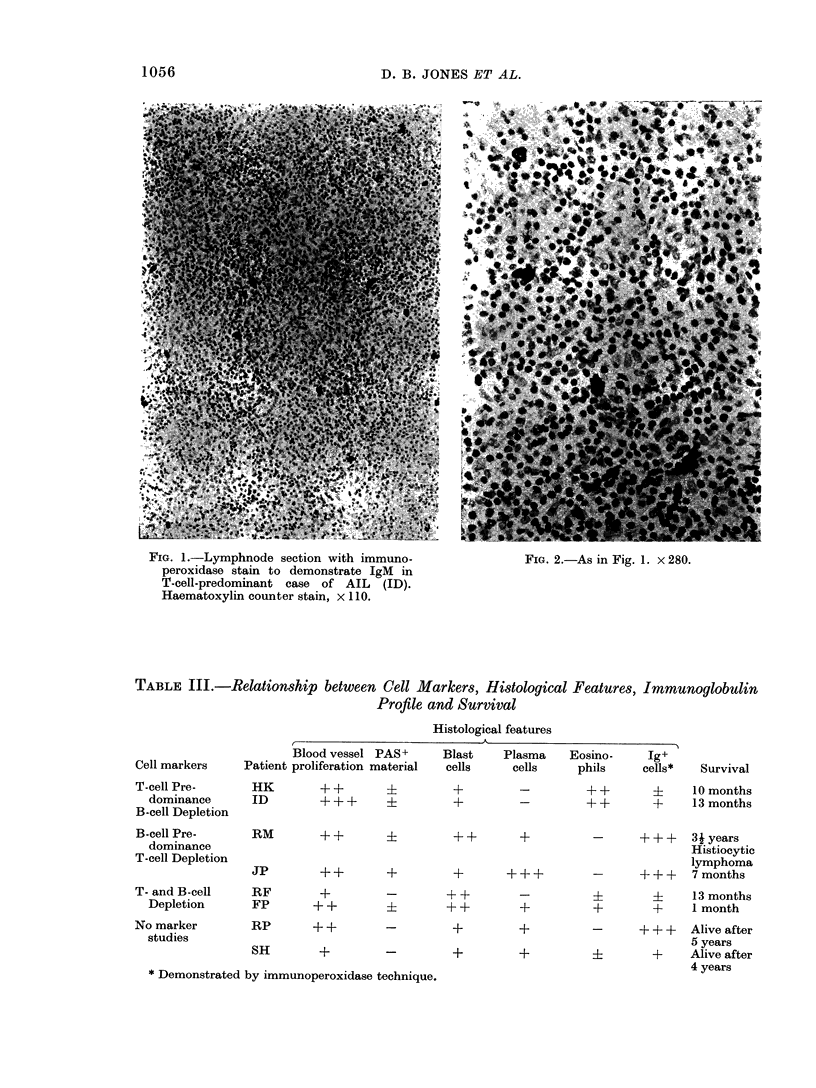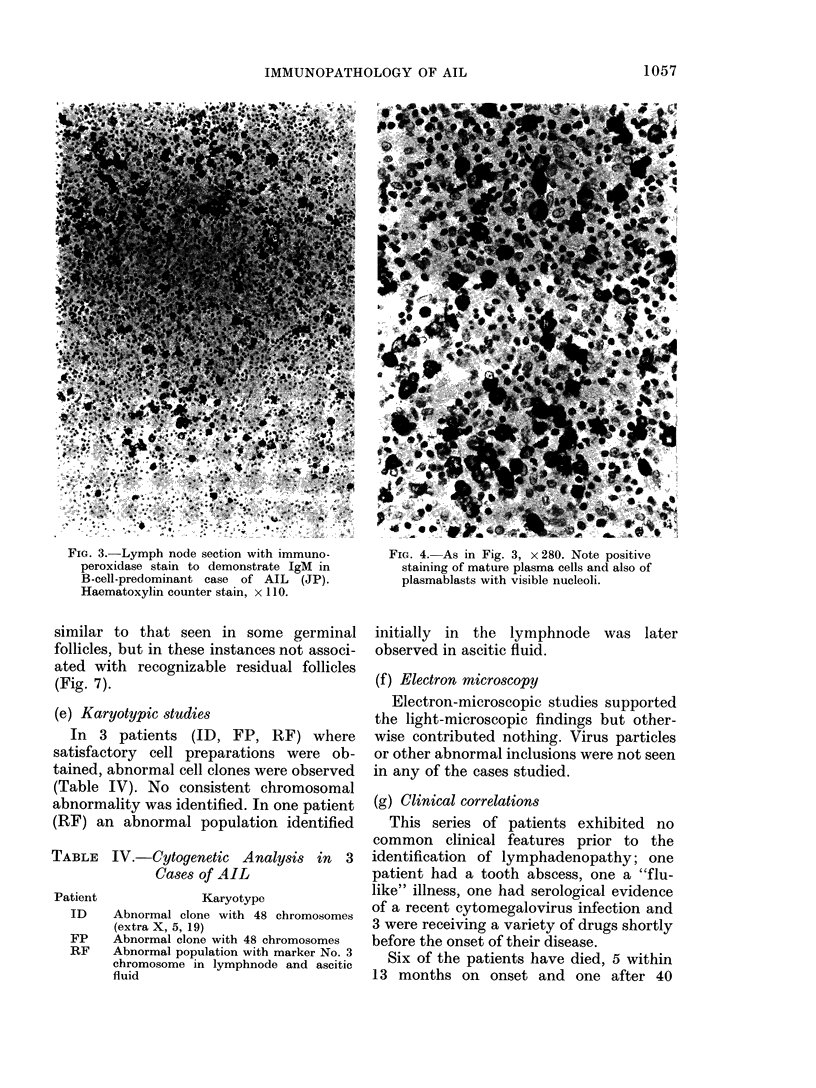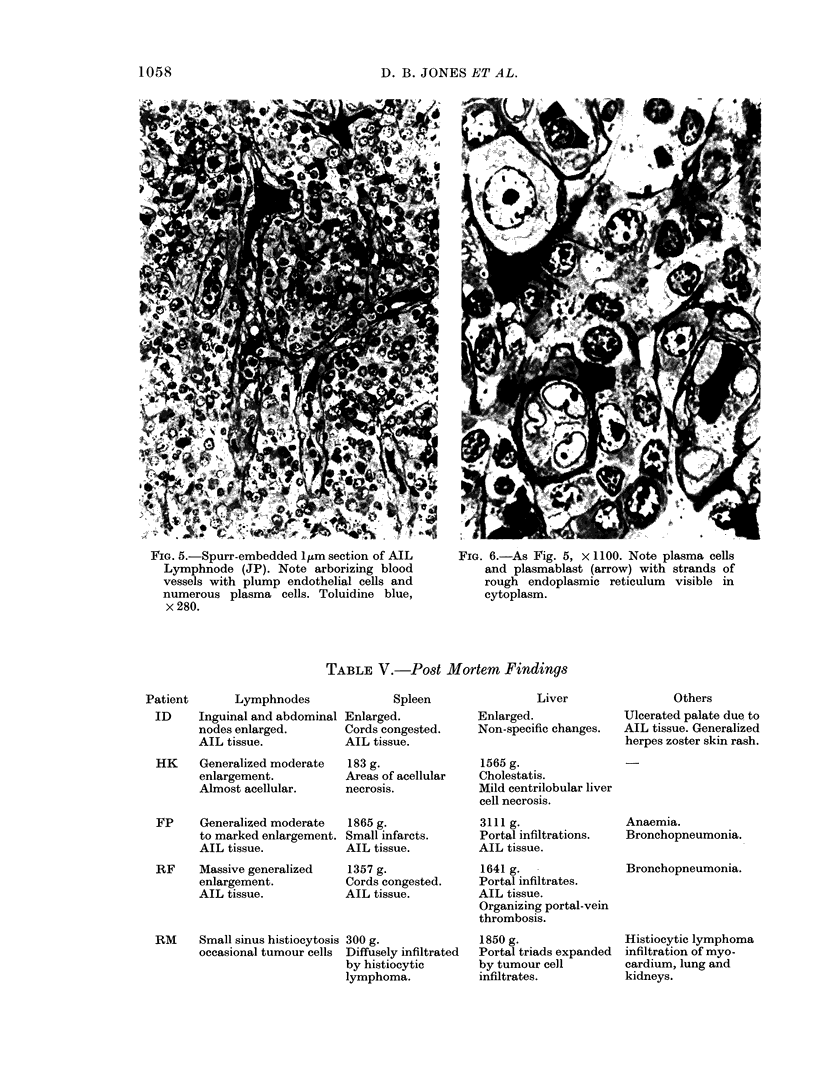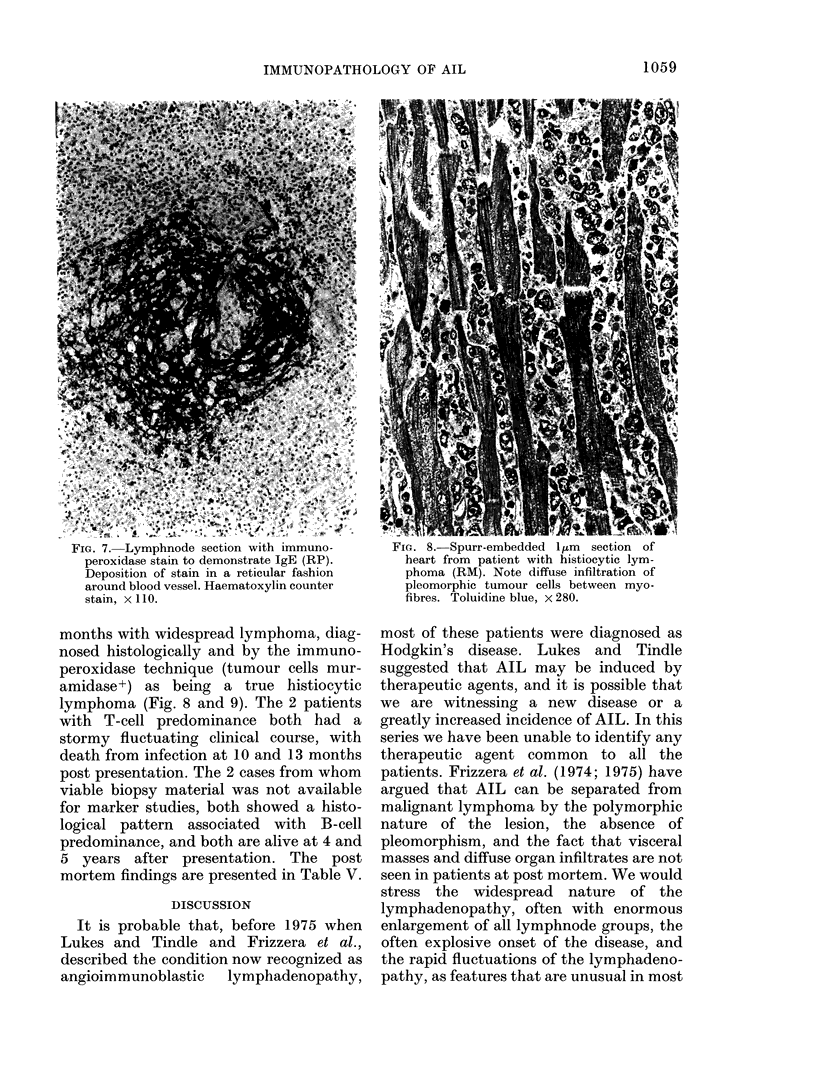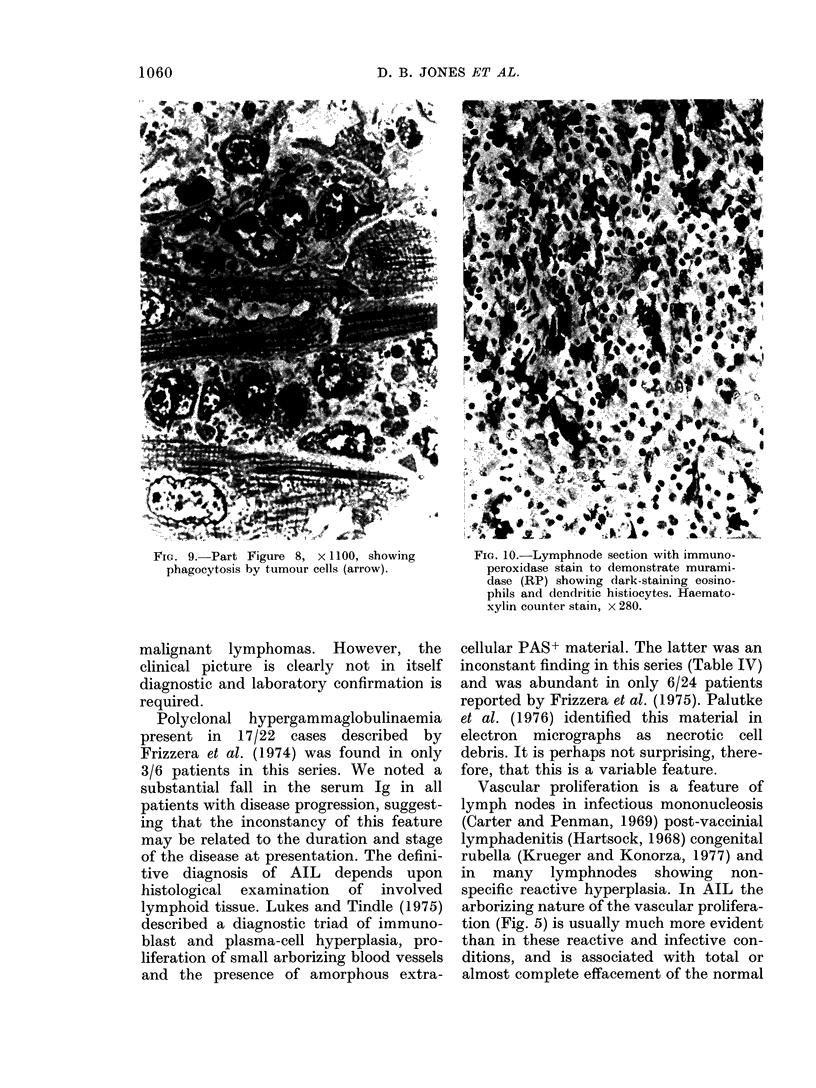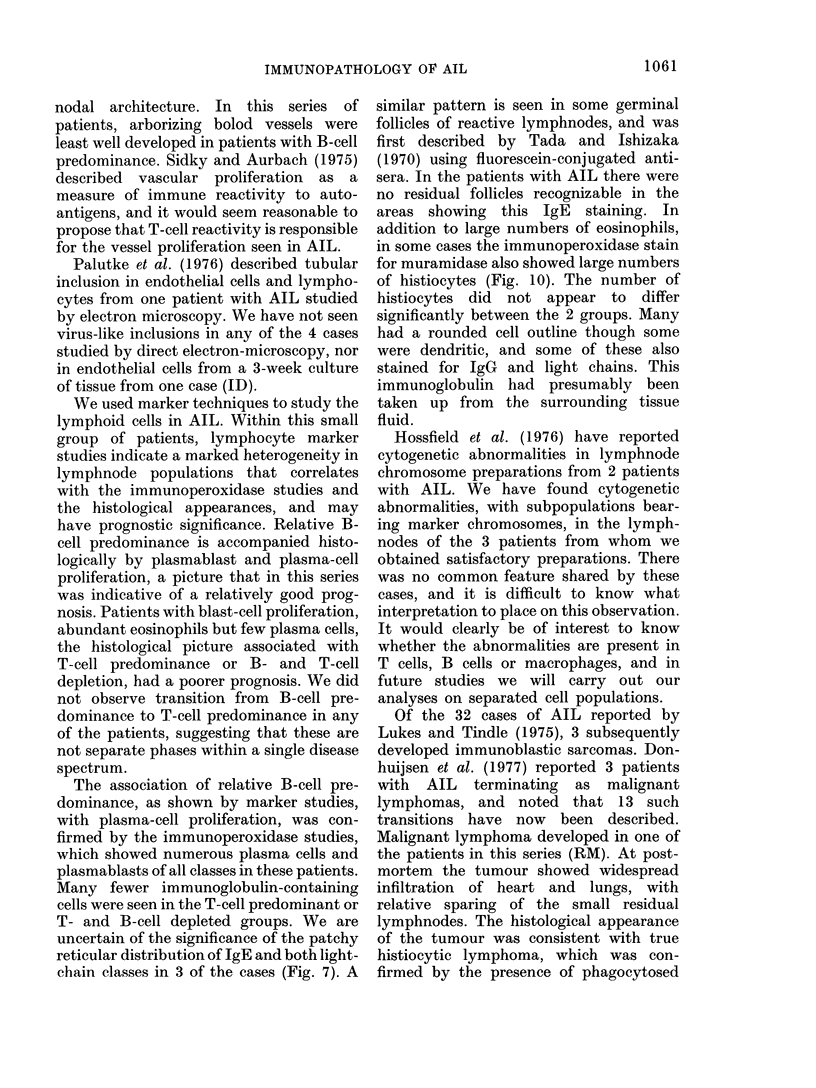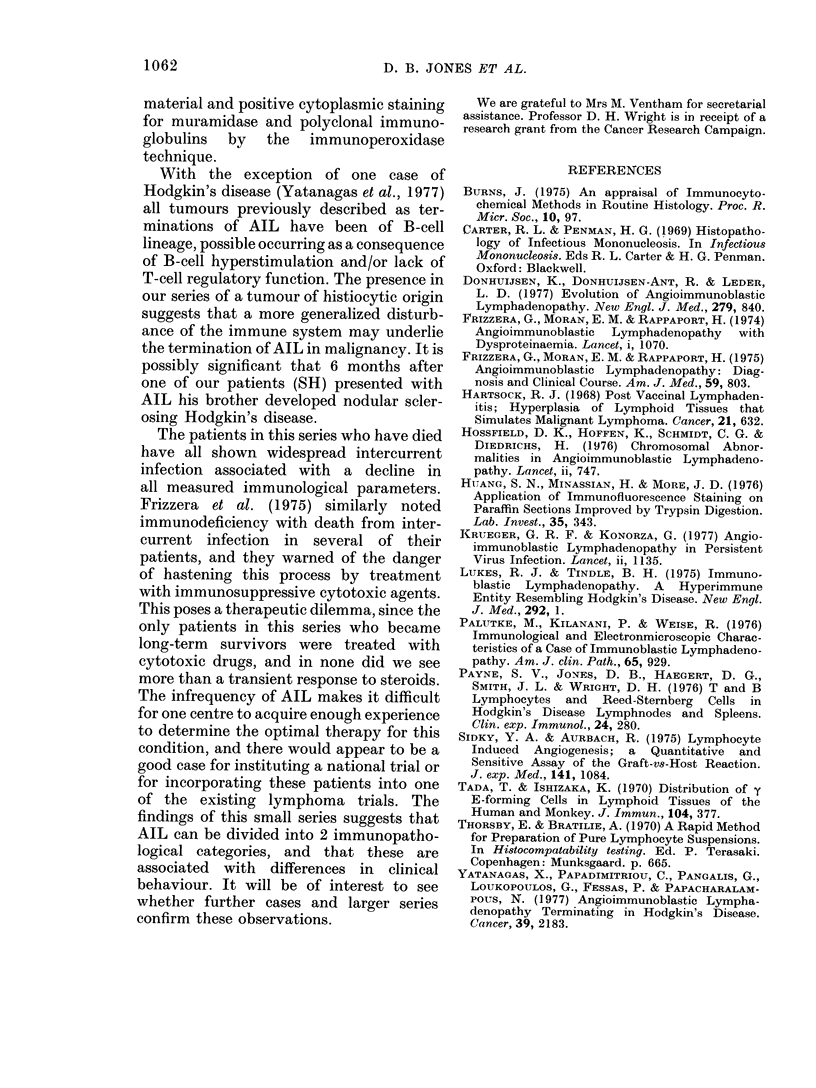# Immunopathology of angioimmunoblastic lymphadenopathy.

**DOI:** 10.1038/bjc.1978.153

**Published:** 1978-06

**Authors:** D. B. Jones, M. Castleden, J. L. Smith, B. L. Mepham, D. H. Wright

## Abstract

**Images:**


Br. J. Cancer (1978) **37**, 1053

### IMMUNOPATHOLOGY OF ANGIOIMMUNOBLASTIC LYMPHADENOPATHY

D. B. JONES*, M. CASTLEDEN†, J. L. SMITH‡, B. L. MEPHAM* AND D. H. WRIGHT*

*From the *University Department of Pathology, †Clinical Pharmacology and ‡Regional Immunology Service, General Hospital, Tremona Road, Southampton*

Received 17 February 1978 Accepted 20 March 1978

**Summary.—Eight patients with angioimmunoblastic lymphadenopathy have been studied by a variety of immunological and pathological techniques. They exhibited a spectrum of immunological reactivities that, in this small series, could be roughly correlated with survival. Those patients with relative B-cell predominance as shown by cell marker studies, histologically showed large numbers of plasma cells, and this pattern was associated in 3 of our patients with a survival of 3 years or more. T-cell predominance or both B- and T-cell depletion was associated histologically with large numbers of blast cells and eosinophils, but with few plasma cells. These patients responded poorly to therapy and had short survival times. One patient with B-cell predominance subsequently died of a histiocytic lymphoma.**

IN 1975 Lukes and Tindle described 32 patients with lymphadenopathy histologically characterized by a proliferation of arborizing small vessels, prominent immunoblastic hyperplasia and amorphous acidophilic, PAS$^+$, interstitial material. They designated this condition as immunoblastic lymphadenopathy, and proposed that it developed as a non-neoplastic hyperimmune proliferation of the B-cell system, involving an exaggeration of lymphocyte transformation to lymphoblasts and plasma cells, triggered by an abnormal hypersensitivity response to therapeutic agents. In 3 of their patients the process evolved into an immunoblastic sarcoma. Frizzera *et al.* (1975) described 24 patients with a syndrome characterized clinically by severe constitutional symptoms, generalized lymphadenopathy, hepatosplenomegaly and polyclonal hypergammaglobulinaemia and with histopathological features similar to those described by Lukes and Tindle. They designated this condition as angioimmunoblastic lymphadenopathy with dysproteinaemia (AILD) and proposed that the various manifestations of this condition are consistent with an autoimmune disorder in which a deficiency of the T-cell regulatory function predisposes to an abnormal proliferation of autoaggressive B lymphocytes. None of their patients progressed to lymphoma. It is probably that this entity is the same as that described by Lukes and Tindle, or at least that there is a considerable overlap in the 2 series.

We have recently seen 8 patients with angioimmunoblastic lymphadenopathy and have been able to undertake immunological studies on fresh lymphnode material from 5 of these. The results presented reveal an immunological heterogeneity correlated with histological features, reflecting either the evolution of the disease with time or the inclusion of spectrum of reactivities in this condition.

### MATERIALS AND METHODS

*Patients.*—Seven of the patients were seen in hospitals in the Southampton District during the period 1973–77 and diagnosed on routine lymphnode biopsy for the investiga-

Reprint requests to Professor D. H. Wright.

tion of lymphadenopathy. One patient was originally diagnosed as mixed-cellularity Hodgkin's disease in 1972, and was identified as AIL in 1976 when his original histological sections were reviewed.

Repeat lymphnode biopsies were obtained fresh from 5 of the 8 patients. Paraffin-embedded sections were stained by haematoxylin and eosin, PAS, methyl green pyronin and Gordon and Sweet's reticulin technique for histological examination.

*Cell suspensions.*—Fresh lymphnode material was finely minced and teased in cold HEPES-buffered RPMI 1640 (Flow Laboratories, Irvine, Scotland), filtered through gauze and layered over Ficoll-Triosil (Thorsby and Bratilie, 1970). Cells collected at the interface were washed ×3 in cold medium and the final pellet resuspended at $2 \times 10^6$ cells/ml in HEPES-buffered medium for cell-surface marker-studies. Peripheral-blood lymphocytes were similarly prepared from venous blood by centrifugation through Ficoll-Triosil.

*Rosette tests.*—Viable mononuclear cell preparations were examined for T lymphocytes by spontaneous sheep-cell rosetting, and for the presence of receptors for C3 and the Fc portion of Ig by rosetting with appropriately sensitized ox red blood cells. Cell suspensions were further stained with a polyvalent rabbit antiserum to human immunoglobulin heavy chains and to $\kappa$ and $\lambda$ light chains. Full details of these tests have been published previously (Payne *et al.*, 1976).

*Immunoperoxidase technique.*—For the demonstration of intracellular immunoglobulins the unlabelled antibody/enzyme method (PAP) was performed on 5$\mu$m paraffin sections, using the basic technique described by Burns (1975) following treatment with 0·1% trypsin (Huang *et al.*, 1976) after blocking endogenous peroxidase activity with methanol/$H_2O_2$. Rabbit antiserum to human A G M D E heavy chains and $\kappa$ and $\lambda$ light chains (Hoechst Pharmaceuticals, Hounslow), rabbit anti-human muramidase (lysozyme), swine anti-rabbit IgG and PAP (Dako; agents Mercia Diagnostics, Watford, Herts) were titrated on control sections and used at their respective optimum titres. Specificity of antisera was established using radial immunodiffusion and immunoelectrophoretic techniques. The replacement of antisera with non-immune rabbit serum and a slide treated with diaminobenzidine reagent alone served as controls.

*Electron microscopy.*—Tissue from 5 cases was processed for electron microscopy, either by immediate fixation in 4% gluteraldehyde in cacodylate buffer at pH 7·2 or by transfer from 10% buffered formol saline to gluteraldehyde for 24 h. Tissue was post-fixed in osmium tetroxide, embedded in Spurr resin and sectioned on a Reichert OMU3 ultramicrotome. Thin sections were stained with lead citrate/uranyl acetate and examined in a Phillips 201 electron microscope.

*Cytogenetics.*—Cytogenetic analysis was performed on dispersed lymphnode cells at the Regional Cytology Laboratory by Dr M. Seabright.

### RESULTS

### (a) *Marker studies*

The results of lymphocyte marker studies performed on viable cells dispersed from lymphnode biopsy material from 5 patients with AIL are presented in Table II. By comparison with control values these patients appear to form a heterogeneous group exhibiting either relative T-cell predominance (ID, HK) relative B-cell predominance (RM, JP) or an apparent depletion of both lymphocyte subsets (RF).

All patients were lymphopenic at presentation or became so during the course of their disease. In 3 patients (HK, FP, RF) the relative percentage of T lymphocytes in the peripheral blood measured by spontaneous sheep-erythrocyte rosetting was below the normal range. With advancing disease and treatment all patients showed a decline in T-cell percentages.

### (b) *Serum studies*

A polyclonal elevation in serum immunoglobulin was observed in 3 patients (ID, RM, JP) an increase not confined to any single immunoglobulin class (Table II). In all patients serum immunoglobulin levels declined with advancing disease.

Urinary paraproteins were not detected in any of the 8 patients studied. Autoantibody activity; directed to thryoid microsomes was detected in only one

TABLE I.—*Clinical Features at Presentation*

| Patient | Age/sex | Lymphadenopathy | Hepatomegaly | Splenomegaly | Skin rash | Fever |
|---|---|---|---|---|---|---|
| SH | 29/M | + | – | – | – | – |
| ID | 46/F | + | – | + | + | + |
| HK | 55/M | + | – | – | – | – |
| FP | 61/M | + | + | + | – | + |
| RM | 66/M | + | + | + | – | – |
| RF | 71/M | + | + | + | – | – |
| RP | 76/M | + | + | + | – | – |
| JP | 84/M | + | + | + | – | + |

TABLE II.—*Surface Marker and Immunochemical Data in AIL*

| Patient | Specimen† | WBC/l (×10⁹) | Lymphs/l (×10⁹) | % Lymphs T | % Lymphs Ig | % Lymphs B/Fc | % Lymphs C3 | Serum immunochemistry (g/l) G | A | M |
|---|---|---|---|---|---|---|---|---|---|---|
| ID | LN | — | — | 74 | 10 | 7 | 2 | | | |
| | BL | 7·5 | 0·53 | 59 | 12 | — | — | 11·2 | 0·6 | 2·5* |
| HK | LN | — | — | 68 | 4 | 9 | 6 | | | |
| | BL | 5·5 | 0·9 | 47 | — | 25 | 21 | 6·6 | 2·4 | 0·8 |
| FP | LN | — | — | — | — | — | — | | | |
| | BL | 0·9 | 0·27 | 45 | 28 | 44 | 60 | 10·2 | 3·8 | 0·4 |
| RM | LN | — | — | 37 | 68 | 36 | 63 | | | |
| | BL | nd | nd | — | — | — | — | 19·7* | 3·4 | 2·8* |
| RF | LN | — | — | 35 | — | 16 | 18 | | | |
| | BL | 4·0 | 1·8 | 32 | 3 | 39 | 14 | 6·4 | 2·4 | 1·5 |
| JP | LN | — | — | 21 | 66 | 74 | 69 | | | |
| | BL | nd | nd | 62 | nd | nd | nd | 19·8* | 4·6* | 0·60 |
| Normal values | LN | — | — | (54 ±12) | (25 ±13) | (9 ±7) | (43 ±12) | | | |
| | BL | (3–9) (limits) | (1–3) (limits) | (60 ±10) | (20 ±5) (mean±range) | (25 ±10) (mean±range) | (25 ±10) | (7·0 –13·0) (limits) | (1·2 –3·5) (limits) | (0·4 –1·3) (limits) |

† BL = Venous Blood taken at presentation.
LN = Viable Lymphnode Cell preparations.
* = Levels significantly raised.

patient (ID) and this patient also exhibited a positive indirect Coomb's test.

(c) *Histopathological correlations*

Marker studies performed on fresh biopsy material correlated with the histological appearance of the lymph nodes (Table III). Patients with a high ratio of T to B cells or T- and B-cell depletion (ID, HK, RF) showed prominent arborising blood vessels, numerous eosinophils, blast cell proliferation but few plasmablasts or plasma cells (Fig. 1 and 2) whereas patients with a high B/T ratio (RM, JP) showed marked plasma-cell and plasmablast proliferation but few or no eosinophils (Fig. 3, 4, 5 and 6).

(d) *Immunoperoxidase*

The immunoperoxidase findings paralleled the histological appearances and marker studies (Table III) in that the 2 patients with B-cell predominance showed large numbers of immunoglobulin containing plasma cells and plasmablasts of all classes including a moderate number of cells staining for IgD and IgE (Fig. 3 and 4). One of the patients on whom marker studies were not done showed large numbers of Ig-containing cells and the other, moderate numbers. $IgG^+$ cells were most frequent, followed by $IgA^+$ and $IgM^+$. In 3 patients (ID, FP, RP) staining with anti-IgE and anti-light-chain sera showed a focal reticular pattern of staining

FIG. 1.—Lymphnode section with immunoperoxidase stain to demonstrate IgM in T-cell-predominant case of AIL (ID). Haematoxylin counter stain, ×110.

FIG. 2.—As in Fig. 1. ×280.

TABLE III.—*Relationship between Cell Markers, Histological Features, Immunoglobulin Profile and Survival*

| Cell markers | Patient | Histological features: Blood vessel proliferation | PAS+ material | Blast cells | Plasma cells | Eosinophils | Ig+ cells* | Survival |
|---|---|---|---|---|---|---|---|---|
| T-cell Predominance B-cell Depletion | HK | ++ | ± | + | − | ++ | ± | 10 months |
| | ID | +++ | ± | + | − | ++ | + | 13 months |
| B-cell Predominance T-cell Depletion | RM | ++ | ± | ++ | + | − | +++ | 3½ years Histiocytic lymphoma |
| | JP | ++ | + | + | +++ | − | +++ | 7 months |
| T- and B-cell Depletion | RF | + | − | ++ | − | ± | ± | 13 months |
| | FP | ++ | ± | ++ | + | + | + | 1 month |
| No marker studies | RP | ++ | − | + | + | − | +++ | Alive after 5 years |
| | SH | + | − | + | + | ± | + | Alive after 4 years |

* Demonstrated by immunoperoxidase technique.

FIG. 3.—Lymph node section with immunoperoxidase stain to demonstrate IgM in B-cell-predominant case of AIL (JP). Haematoxylin counter stain, ×110.

FIG. 4.—As in Fig. 3, ×280. Note positive staining of mature plasma cells and also of plasmablasts with visible nucleoli.

similar to that seen in some germinal follicles, but in these instances not associated with recognizable residual follicles (Fig. 7).

(e) *Karyotypic studies*

In 3 patients (ID, FP, RF) where satisfactory cell preparations were obtained, abnormal cell clones were observed (Table IV). No consistent chromosomal abnormality was identified. In one patient (RF) an abnormal population identified initially in the lymphnode was later observed in ascitic fluid.

TABLE IV.—*Cytogenetic Analysis in 3 Cases of AIL*

| Patient | Karyotype |
|---|---|
| ID | Abnormal clone with 48 chromosomes (extra X, 5, 19) |
| FP | Abnormal clone with 48 chromosomes |
| RF | Abnormal population with marker No. 3 chromosome in lymphnode and ascitic fluid |

(f) *Electron microscopy*

Electron-microscopic studies supported the light-microscopic findings but otherwise contributed nothing. Virus particles or other abnormal inclusions were not seen in any of the cases studied.

(g) *Clinical correlations*

This series of patients exhibited no common clinical features prior to the identification of lymphadenopathy; one patient had a tooth abscess, one a "flu-like" illness, one had serological evidence of a recent cytomegalovirus infection and 3 were receiving a variety of drugs shortly before the onset of their disease.

Six of the patients have died, 5 within 13 months on onset and one after 40

FIG. 5.—Spurr-embedded 1μm section of AIL Lymphnode (JP). Note arborizing blood vessels with plump endothelial cells and numerous plasma cells. Toluidine blue, ×280.

FIG. 6.—As Fig. 5, ×1100. Note plasma cells and plasmablast (arrow) with strands of rough endoplasmic reticulum visible in cytoplasm.

TABLE V.—*Post Mortem Findings*

| Patient | Lymphnodes | Spleen | Liver | Others |
|---|---|---|---|---|
| ID | Inguinal and abdominal nodes enlarged. AIL tissue. | Enlarged. Cords congested. AIL tissue. | Enlarged. Non-specific changes. | Ulcerated palate due to AIL tissue. Generalized herpes zoster skin rash. |
| HK | Generalized moderate enlargement. Almost acellular. | 183 g. Areas of acellular necrosis. | 1565 g. Cholestatis. Mild centrilobular liver cell necrosis. | — |
| FP | Generalized moderate to marked enlargement. AIL tissue. | 1865 g. Small infarcts. AIL tissue. | 3111 g. Portal infiltrations. AIL tissue. | Anaemia. Bronchopneumonia. |
| RF | Massive generalized enlargement. AIL tissue. | 1357 g. Cords congested. AIL tissue. | 1641 g. Portal infiltrates. AIL tissue. Organizing portal-vein thrombosis. | Bronchopneumonia. |
| RM | Small sinus histiocytosis occasional tumour cells | 300 g. Diffusely infiltrated by histiocytic lymphoma. | 1850 g. Portal triads expanded by tumour cell infiltrates. | Histiocytic lymphoma infiltration of myocardium, lung and kidneys. |

FIG. 7.—Lymphnode section with immunoperoxidase stain to demonstrate IgE (RP). Deposition of stain in a reticular fashion around blood vessel. Haematoxylin counter stain, ×110.

FIG. 8.—Spurr-embedded 1μm section of heart from patient with histiocytic lymphoma (RM). Note diffuse infiltration of pleomorphic tumour cells between myofibres. Toluidine blue, ×280.

months with widespread lymphoma, diagnosed histologically and by the immunoperoxidase technique (tumour cells muramidase$^+$) as being a true histiocytic lymphoma (Fig. 8 and 9). The 2 patients with T-cell predominance both had a stormy fluctuating clinical course, with death from infection at 10 and 13 months post presentation. The 2 cases from whom viable biopsy material was not available for marker studies, both showed a histological pattern associated with B-cell predominance, and both are alive at 4 and 5 years after presentation. The post mortem findings are presented in Table V.

### DISCUSSION

It is probable that, before 1975 when Lukes and Tindle and Frizzera *et al.*, described the condition now recognized as angioimmunoblastic lymphadenopathy, most of these patients were diagnosed as Hodgkin's disease. Lukes and Tindle suggested that AIL may be induced by therapeutic agents, and it is possible that we are witnessing a new disease or a greatly increased incidence of AIL. In this series we have been unable to identify any therapeutic agent common to all the patients. Frizzera *et al.* (1974; 1975) have argued that AIL can be separated from malignant lymphoma by the polymorphic nature of the lesion, the absence of pleomorphism, and the fact that visceral masses and diffuse organ infiltrates are not seen in patients at post mortem. We would stress the widespread nature of the lymphadenopathy, often with enormous enlargement of all lymphnode groups, the often explosive onset of the disease, and the rapid fluctuations of the lymphadenopathy, as features that are unusual in most

Fig. 9.—Part Figure 8, ×1100, showing phagocytosis by tumour cells (arrow).

Fig. 10.—Lymphnode section with immunoperoxidase stain to demonstrate muramidase (RP) showing dark-staining eosinophils and dendritic histiocytes. Haematoxylin counter stain, ×280.

malignant lymphomas. However, the clinical picture is clearly not in itself diagnostic and laboratory confirmation is required.

Polyclonal hypergammaglobulinaemia present in 17/22 cases described by Frizzera *et al.* (1974) was found in only 3/6 patients in this series. We noted a substantial fall in the serum Ig in all patients with disease progression, suggesting that the inconstancy of this feature may be related to the duration and stage of the disease at presentation. The definitive diagnosis of AIL depends upon histological examination of involved lymphoid tissue. Lukes and Tindle (1975) described a diagnostic triad of immunoblast and plasma-cell hyperplasia, proliferation of small arborizing blood vessels and the presence of amorphous extracellular PAS$^+$ material. The latter was an inconstant finding in this series (Table IV) and was abundant in only 6/24 patients reported by Frizzera *et al.* (1975). Palutke *et al.* (1976) identified this material in electron micrographs as necrotic cell debris. It is perhaps not surprising, therefore, that this is a variable feature.

Vascular proliferation is a feature of lymph nodes in infectious mononucleosis (Carter and Penman, 1969) post-vaccinial lymphadenitis (Hartsock, 1968) congenital rubella (Krueger and Konorza, 1977) and in many lymphnodes showing nonspecific reactive hyperplasia. In AIL the arborizing nature of the vascular proliferation (Fig. 5) is usually much more evident than in these reactive and infective conditions, and is associated with total or almost complete effacement of the normal nodal architecture. In this series of patients, arborizing bolod vessels were least well developed in patients with B-cell predominance. Sidky and Aurbach (1975) described vascular proliferation as a measure of immune reactivity to auto-antigens, and it would seem reasonable to propose that T-cell reactivity is responsible for the vessel proliferation seen in AIL.

Palutke *et al.* (1976) described tubular inclusion in endothelial cells and lymphocytes from one patient with AIL studied by electron microscopy. We have not seen virus-like inclusions in any of the 4 cases studied by direct electron-microscopy, nor in endothelial cells from a 3-week culture of tissue from one case (ID).

We used marker techniques to study the lymphoid cells in AIL. Within this small group of patients, lymphocyte marker studies indicate a marked heterogeneity in lymphnode populations that correlates with the immunoperoxidase studies and the histological appearances, and may have prognostic significance. Relative B-cell predominance is accompanied histologically by plasmablast and plasma-cell proliferation, a picture that in this series was indicative of a relatively good prognosis. Patients with blast-cell proliferation, abundant eosinophils but few plasma cells, the histological picture associated with T-cell predominance or B- and T-cell depletion, had a poorer prognosis. We did not observe transition from B-cell predominance to T-cell predominance in any of the patients, suggesting that these are not separate phases within a single disease spectrum.

The association of relative B-cell predominance, as shown by marker studies, with plasma-cell proliferation, was confirmed by the immunoperoxidase studies, which showed numerous plasma cells and plasmablasts of all classes in these patients. Many fewer immunoglobulin-containing cells were seen in the T-cell predominant or T- and B-cell depleted groups. We are uncertain of the significance of the patchy reticular distribution of IgE and both light-chain classes in 3 of the cases (Fig. 7). A similar pattern is seen in some germinal follicles of reactive lymphnodes, and was first described by Tada and Ishizaka (1970) using fluorescein-conjugated antisera. In the patients with AIL there were no residual follicles recognizable in the areas showing this IgE staining. In addition to large numbers of eosinophils, in some cases the immunoperoxidase stain for muramidase also showed large numbers of histiocytes (Fig. 10). The number of histiocytes did not appear to differ significantly between the 2 groups. Many had a rounded cell outline though some were dendritic, and some of these also stained for IgG and light chains. This immunoglobulin had presumably been taken up from the surrounding tissue fluid.

Hossfield *et al.* (1976) have reported cytogenetic abnormalities in lymphnode chromosome preparations from 2 patients with AIL. We have found cytogenetic abnormalities, with subpopulations bearing marker chromosomes, in the lymphnodes of the 3 patients from whom we obtained satisfactory preparations. There was no common feature shared by these cases, and it is difficult to know what interpretation to place on this observation. It would clearly be of interest to know whether the abnormalities are present in T cells, B cells or macrophages, and in future studies we will carry out our analyses on separated cell populations.

Of the 32 cases of AIL reported by Lukes and Tindle (1975), 3 subsequently developed immunoblastic sarcomas. Donhuijsen *et al.* (1977) reported 3 patients with AIL terminating as malignant lymphomas, and noted that 13 such transitions have now been described. Malignant lymphoma developed in one of the patients in this series (RM). At postmortem the tumour showed widespread infiltration of heart and lungs, with relative sparing of the small residual lymphnodes. The histological appearance of the tumour was consistent with true histiocytic lymphoma, which was confirmed by the presence of phagocytosed material and positive cytoplasmic staining for muramidase and polyclonal immunoglobulins by the immunoperoxidase technique.

With the exception of one case of Hodgkin's disease (Yatanagas *et al.*, 1977) all tumours previously described as terminations of AIL have been of B-cell lineage, possible occurring as a consequence of B-cell hyperstimulation and/or lack of T-cell regulatory function. The presence in our series of a tumour of histiocytic origin suggests that a more generalized disturbance of the immune system may underlie the termination of AIL in malignancy. It is possibly significant that 6 months after one of our patients (SH) presented with AIL his brother developed nodular sclerosing Hodgkin's disease.

The patients in this series who have died have all shown widespread intercurrent infection associated with a decline in all measured immunological parameters. Frizzera *et al.* (1975) similarly noted immunodeficiency with death from intercurrent infection in several of their patients, and they warned of the danger of hastening this process by treatment with immunosuppressive cytotoxic agents. This poses a therapeutic dilemma, since the only patients in this series who became long-term survivors were treated with cytotoxic drugs, and in none did we see more than a transient response to steroids. The infrequency of AIL makes it difficult for one centre to acquire enough experience to determine the optimal therapy for this condition, and there would appear to be a good case for instituting a national trial or for incorporating these patients into one of the existing lymphoma trials. The findings of this small series suggests that AIL can be divided into 2 immunopathological categories, and that these are associated with differences in clinical behaviour. It will be of interest to see whether further cases and larger series confirm these observations.

We are grateful to Mrs M. Ventham for secretarial assistance. Professor D. H. Wright is in receipt of a research grant from the Cancer Research Campaign.

REFERENCES

BURNS, J. (1975) An appraisal of Immunocytochemical Methods in Routine Histology. *Proc. R. Micr. Soc.*, **10**, 97.

CARTER, R. L. & PENMAN, H. G. (1969) Histopathology of Infectious Mononucleosis. In *Infectious Mononucleosis*. Eds R. L. Carter & H. G. Penman. Oxford: Blackwell.

DONHUIJSEN, K., DONHUIJSEN-ANT, R. & LEDER, L. D. (1977) Evolution of Angioimmunoblastic Lymphadenopathy. *New Engl. J. Med.*, **279**, 840.

FRIZZERA, G., MORAN, E. M. & RAPPAPORT, H. (1974) Angioimmunoblastic Lymphadenopathy with Dysproteinaemia. *Lancet*, i, 1070.

FRIZZERA, G., MORAN, E. M. & RAPPAPORT, H. (1975) Angioimmunoblastic Lymphadenopathy: Diagnosis and Clinical Course. *Am. J. Med.*, **59**, 803.

HARTSOCK, R. J. (1968) Post Vaccinal Lymphadenitis; Hyperplasia of Lymphoid Tissues that Simulates Malignant Lymphoma. *Cancer*, **21**, 632.

HOSSFIELD, D. K., HOFFEN, K., SCHMIDT, C. G. & DIEDRICHS, H. (1976) Chromosomal Abnormalities in Angioimmunoblastic Lymphadenopathy. *Lancet*, ii, 747.

HUANG, S. N., MINASSIAN, H. & MORE, J. D. (1976) Application of Immunofluorescence Staining on Paraffin Sections Improved by Trypsin Digestion. *Lab. Invest.*, **35**, 343.

KRUEGER, G. R. F. & KONORZA, G. (1977) Angioimmunoblastic Lymphadenopathy in Persistent Virus Infection. *Lancet*, ii, 1135.

LUKES, R. J. & TINDLE, B. H. (1975) Immunoblastic Lymphadenopathy. A Hyperimmune Entity Resembling Hodgkin's Disease. *New Engl. J. Med.*, **292**, 1.

PALUTKE, M., KILANANI, P. & WEISE, R. (1976) Immunological and Electronmicroscopic Characteristics of a Case of Immunoblastic Lymphadenopathy. *Am. J. clin. Path.*, **65**, 929.

PAYNE, S. V., JONES, D. B., HAEGERT, D. G., SMITH, J. L. & WRIGHT, D. H. (1976) T and B Lymphocytes and Reed-Sternberg Cells in Hodgkin's Disease Lymphnodes and Spleens. *Clin. exp. Immunol.*, **24**, 280.

SIDKY, Y. A. & AURBACH, R. (1975) Lymphocyte Induced Angiogenesis; a Quantitative and Sensitive Assay of the Graft-*vs*-Host Reaction. *J. exp. Med.*, **141**, 1084.

TADA, T. & ISHIZAKA, K. (1970) Distribution of γ E-forming Cells in Lymphoid Tissues of the Human and Monkey. *J. Immun.*, **104**, 377.

THORSBY, E. & BRATILIE, A. (1970) A Rapid Method for Preparation of Pure Lymphocyte Suspensions. In *Histocompatability testing*. Ed. P. Terasaki. Copenhagen: Munksgaard. p. 665.

YATANAGAS, X., PAPADIMITRIOU, C., PANGALIS, G., LOUKOPOULOS, G., FESSAS, P. & PAPACHARALAMPOUS, N. (1977) Angioimmunoblastic Lymphadenopathy Terminating in Hodgkin's Disease. *Cancer*, **39**, 2183.